# Identifying the Barriers to Acceptance of Blockchain-Based Patient-Centric Data Management Systems in Healthcare

**DOI:** 10.3390/healthcare12030345

**Published:** 2024-01-30

**Authors:** Ibrahim Mutambik, John Lee, Abdullah Almuqrin, Zahyah H. Alharbi

**Affiliations:** 1 Department of Information Science, College of Humanities and Social Sciences, King Saud University, P.O. Box 11451, Riyadh 11437, Saudi Arabia; aalmogren@ksu.edu.sa; 2 School of Informatics, The University of Edinburgh, 10 Crichton St., Edinburgh EH8 9AB, UK; john.lee@ed.ac.uk; 3 Department of Management Information Systems, College of Business Administration, King Saud University, P.O. Box 28095, Riyadh 11437, Saudi Arabia; zalharbi@ksu.edu.sa

**Keywords:** blockchain, blockchain barriers, blockchain in healthcare, future of healthcare blockchain, GCC countries

## Abstract

A number of recent studies have shown that wastage and inefficiency are a significant problem in all global healthcare systems. One initiative that could radically improve the operational efficiency of health systems is to make a paradigm shift in data ownership—that is, to transition such systems to a patient-centric model of data management by deploying blockchain technology. Such a development would not only make an economic impact, by radically cutting wastage, but would deliver significant social benefits by improving patient outcomes and satisfaction. However, a blockchain-based solution presents considerable challenges. This research seeks to understand the principal factors, which act as barriers to the acceptance of a blockchain-based patient-centric data management infrastructure, in the healthcare systems of the GCC (Gulf Cooperation Council) countries. The study represents an addition to the current literature by examining the perspectives and views of healthcare professionals and users. This approach is rare within this subject area, and is identified in existing systematic reviews as a research gap: a qualitative investigation of motivations and attitudes among these groups is a critical need. The results of the study identified 12 key barriers to the acceptance of blockchain infrastructures, thereby adding to our understanding of the challenges that need to be overcome in order to benefit from this relatively recent technology. The research is expected to be of use to healthcare authorities in planning a way forward for system improvement, particularly in terms of successfully introducing patient-centric systems.

## 1. Introduction

In most countries today, the efficiency of healthcare systems is critical. Yet few, if any, nations can claim that there is not a high level of wastage inherent within their existing health infrastructure [1]. According to a 2023 report by the Peter G. Peterson Foundation, for example, as much as 25% of healthcare expenditure in the US alone is considered wasteful, which amounted, in 2021, to some USD 1.2 trillion annually [2,3], while various other studies have found that similar problems beset all OECD countries [4,5].

One of the principal reasons for this inefficiency lies in the complex and multi-dimensional interactions between various (healthcare system) stakeholders, ranging from primary care actors (clinical practitioners) and the patients themselves, to hospitals and national governments [4,5]. The result, without exception, has been the deployment of technology in a disparate and non-integrated way, by using different digital platforms for different parts of the system. This leads to a number of serious issues, resulting from an inability to find and exchange patient data across multiple health providers and related organisations [6], including increased costs, high error rates, knowledge mismanagement, poor quality of care [7] and higher mortality rates [6]. Yet, creating an integrated system retrospectively has proved a formidable challenge.

In the past few years, however, a powerful, cost-efficient, and—in principle—technically viable solution has emerged: blockchain. This is essentially a form of distributed (decentralised) database, which means that the database on which transactions are recorded is shared by several participants (called nodes) in the blockchain. Such a decentralised infrastructure allows for a faster, more secure transaction process, which all participants can trust.

The evidence is clear [4,8,9] that blockchain offers a major opportunity to develop healthcare systems across the world to better meet evolving societal and economic development goals. However, while the technology is being increasingly implemented [6,10,11,12], it is, according to the World Economic Forum, still “massively under-utilised in global healthcare” [12]. There have been a number of studies that have investigated why this is the case. One such study, for example, highlighted the importance of perceived security in encouraging the adoption of blockchain [13,14,15], while others have found that a government’s published position on the technology has a significant effect on adoption levels [13,14,16].

These studies have contributed significantly to our knowledge, indicating that acceptance of the technology by various stakeholders remains an impediment to adoption. However, these studies have all had a relatively narrow focus on specific issues. There are few studies that have examined the barriers to blockchain adoption by considering a wider range of general factors, such as administrative challenges, usability issues, future-proofing, and regulatory frameworks, from the direct perspective of professionals involved, either internally or externally, in the healthcare system [17,18,19]. Existing systematic reviews have also identified a research gap in this area. For example, Tandon et al. [20] conducted an extensive systematic review of the literature on blockchain in healthcare specifically, and noted among their recommendations:

*based on the gaps explicated from the review, implications arise for the need to broader disciplinary and particularly methodological coverage of the research to advance the current understanding of the field. For example, research based on survey methodology or interviews can enhance the understanding of e.g., the challenges and barriers inhibiting user adoption*.

(p. 20)

Similarly, Yang et al. [21], addressing a business context but identifying issues that arise equally in healthcare, observe that

*there is also a lack of empirical studies examining the incentives leading business organizations to invest in and adopt blockchain technology. Indeed, knowledge about the reasons for adopting and using blockchain technology in private and public organizations is rather scarce. We suggest that future studies investigate the motivations associated with blockchain adoption and how these motivations influence how blockchain initiatives are implemented and managed in companies*.

(p. 4480)

Insights into the nature of barriers and inhibitions to user adoption that arise from the attitudes, motivations, and understandings of participants in the specific area of activity are exactly the motivation behind this study, as such insights will support healthcare providers in providing more holistic and efficient systems, yielding a broad range of societal benefits.

This study aims to address this issue by identifying key challenges/barriers to the acceptance of a blockchain-based patient-centric data management system, as a first step towards the development of effective solutions. To achieve this, this study analysed (using thematic analysis) the data collected from semi-structured interviews with 30 professionals working in the healthcare systems of the countries of the Gulf Cooperation Council (GCC). This study explored the research question (see below) across the GCC region, as opposed to a single country, for a number of reasons. Principal among these reasons was the fact that the GCC countries face several similar challenges in the healthcare field, such as a disease burden that includes communicable and non-communicable diseases, mental health issues, and accidental injuries. Addressing this issue will require an innovative and cost-effective strategy [22]. Given that the GCC countries each have government-funded healthcare models, and that the GCC Charter, formulated in 1981, states that the region’s nations should ‘coordinate and integrate’ on joint economic and social ventures [23], it was decided that the study could meaningfully and usefully encompass all nations in the region. As the GCC countries share many cultural, social, and economic characteristics [22,23], the results of the study are expected to be broadly generalisable, though with some limitations. This will be discussed further at a later stage in this report.

A qualitative approach was adopted, as implied by Tandon et al. [20], in order to gain informed, expert opinion which reflects the true, on-the-ground problems that face healthcare providers in implementing a blockchain approach to healthcare infrastructure. This is a crucial precursor to potential future quantitative research. In particular, the paper seeks to provide insights related to the research question:

RQ: What are the main user acceptance barriers to adopting blockchain-based patient-centric data management systems in the GCC healthcare sector?

This paper is structured as follows. Section 2 provides a review of the background, in terms of blockchain-based patient-centric data management systems in healthcare, against which we construct our study. Following this, the research methodology, results, and discussion are presented. Lastly, the conclusions of the study are provided.

## 2. Theoretical Background

There are conflicting claims in the existing literature about the potential benefits that could be delivered by the adoption of blockchain-based ICT solutions in healthcare environments. Many researchers make broad and substantial claims for these benefits. These range from improved data and record management [24,25] to more effective containment of pandemic instances, such as COVID-19 [26,27]. Further, there exist several meta-studies of the blockchain for healthcare development literature, which identifies a wide range of potential use cases for the technology. In general, however, these use cases can be grouped into three main areas: clinical trial improvement, pharmaceutical supply chains, and data ownership (specifically, patient-centred data management systems). We will briefly discuss these in turn.

According to research in [28], the cost of clinical trials approximately doubled between 2008 and 2019. There are a number of reasons for this, such as the inflationary cost of materials, the increasing complexity of regulations, and higher costs associated with collecting, managing, and analysing data. Some of these issues could be entirely eliminated through the implementation of blockchain technology. In fact, blockchain-based systems could effectively revolutionise clinical trial management, by enabling the automation of aspects such as site pre-screening, protocol approvals (such as patient consent, etc.), participant enrolment and monitoring, data collection and compliance, and analysis and reporting [29].

Automating these aspects of clinical trials in a secure, trusted, transparent, and auditable way would radically improve the efficiency of the process. The savings, in terms of both cost and time, could be invested in improving patient care and the development of innovations that would move the healthcare industry forward [30].

There are claims that supply chains, too, could be radically improved through the use of blockchain. Consider, for example, prescription drugs. These are, according to the US National Library of Medicine, the largest counterfeit market in the world, estimated to be worth up to USD 432 billion [31,32]. The WHO (World Health Organisation) reports [33] that up to 10% of medical products worldwide are counterfeit or substandard [34,35], resulting in the death, globally, of more than a million patients every year due to counterfeit pharmaceuticals.

The probability of totally eliminating such criminal activity in the short term is low. However, it is perfectly realistic to reduce the level of crime significantly, through better monitoring and control of supply chains. It is argued that this can be achieved through the use of blockchain. ID (identification) markers can embedded on all products, facilitating full and accurate accountability at every stage of the supply chain by using smart contracts, which are programs stored on blockchain that execute automatically when predetermined conditions are met [6], allowing stakeholders to resolve issues concerning product provenance directly between themselves, without the need for unreliable middlemen [36]. This makes the supply chain not merely less vulnerable to corruption, but also more cost efficient.

The third area where it is argued that blockchain could deliver major benefits is by changing the current model of data ownership to patient-centric data management systems. By using blockchain, the need for third-party data management could be eliminated, allowing individuals to have more control over their medical information through specific apps. This has several significant benefits, such as allowing patients to more easily access test results and medication lists, manage appointments, and contact health and care staff when needed [6]. Such an approach would not merely improve patient outcomes, but would significantly and positively impact system efficiency in terms of the use of resources. In fact, if properly implemented, patient-centric data management has the potential to produce the best possible outcomes in a healthcare context [6,37,38]. For these reasons, patient-centric systems are considered by many to be the ‘holy grail’ of healthcare [39], offering a full restructuring of health systems [6,37,38] and delivering a wide range of economic and social benefits.

While there are several important studies that examine the possibility of blockchain as a basis for patient-centric models [6,11,37], these are all proposals based on theoretical constructs or existing technical knowledge; they are in several respects controversial and do not address the question of whether the technology will be regarded as acceptable from the perspectives of specific and relevant professionals. The present study is different in that it addresses this key aspect.

While many benefits of using blockchain in the healthcare sector are widely proclaimed, a range of factors combine to make the adoption of the technology less straightforward and slower than proponents would like [6,40,41]. One of these factors is that blockchain is still considered to be in its relatively early stages of development, and some significant challenges remain concerning privacy, security and scalability [42]. Another challenge that would need to be overcome, before blockchain can be successfully implemented in healthcare on a wide scale, is the large volume of data concerned. According to a 2020 OECD report [4], storing full electronic medical records or genetic data on blockchain would be costly and inefficient because of the constraints of replicating the blockchain across every network node [43], which undermines the potential of blockchain as a basis for patient-centric data management. The challenges of using very large volumes of data on blockchain have also been identified in other contexts, such as supply chains [6,44,45], which have to be set against the potential advantages of blockchain, such as combatting counterfeiting in this area.

Other problems which arise in the implementation of blockchain include “a lack of standardisation, accessibility, ownership, and change management” [46]. Possibly one of the most significant issues, however, is the need for interoperability between different blockchains. This is because healthcare infrastructures are complex, and would typically involve a number of different blockchain ecosystems. These ecosystems would need to talk seamlessly to each other. However, while interoperability is a significant challenge, viable solutions are beginning to emerge [6,44].

The challenges described above have not completely prevented the adoption of blockchain in healthcare. In fact, the technology is beginning to find acceptance in healthcare environments for a range of administrative and clinical use cases, such as the management of medical records, remote patient monitoring, pharmaceutical supply chains, insurance claims, and data analytics [47]. Yet, as already noted, the proclaimed potential of blockchain technology is far from being realised. This is because one of the most important areas in which blockchain can contribute to social development by helping to revolutionise healthcare is in the third category mentioned above: developing patient-centred data management systems. While there have been some attempts to build patient-centric systems, most have not been as successful as originally hoped [48]. The reasons for this lack of success are unclear, though there is some evidence that inhibiting factors include concerns—by patients, administrators, and clinical staff—over data confidentiality, integrity, and availability [49]. Although there have been a few other studies of the lack of success of similar blockchain projects in the education sector, these have either focused on a specific, pre-identified, adoption barrier [50,51] or have been metastudies that address difficulties only at a very general level [52]. They therefore shed little light on the issues that face the healthcare sector, in terms of blockchain adoption, and specifically the challenges involved in delivering patient-centric systems.

It is this gap in knowledge that this paper attempts to fill. It aims to better understand the challenges that health authorities must overcome if blockchain solutions are to be successful at scale in creating patient-centred data management models. We deploy a qualitative methodology that collects data from a range of stakeholders—administrators, clinical staff, and patients—and analyse these data to identify key issues. While it is true that there is a considerable number of papers on blockchain, most focus narrowly on potential benefits [25,29,53,54]. There are few studies that deploy a qualitative methodology to explore the concerns of the full base of stakeholders. This paper aims to address this.

## 3. Research Method

The aim of this research is to identify the principal barriers to the acceptance of blockchain in healthcare. In particular, we seek to gain insights into acceptance barriers that inhibit the adoption of blockchain-based patient-centric data management systems in the GCC. While there is likely to be a range of significant barriers to adoption in any healthcare environment, there are several aspects of the GCC context (as they share a similar culture and society) that may produce unique and specific barriers. These include, in particular:

- Cultural Factors: Most GCC healthcare systems will use their own unique and familiar record-keeping and data-sharing practices. There may therefore be a strong resistance to disrupting these processes.
- Regulatory Environment: Blockchain technology may not easily align with the regulatory frameworks of the GCC, particularly in terms of data protection and cross-border data exchange.
- Technological Readiness: Blockchain systems may require technical infrastructures and levels of digital literacy among the (staff) population which may not yet exist, and will take significant time to develop.
- Distrust: As a new technology, blockchain may engender distrust in healthcare professionals and patients in the GCC, which may be difficult to overcome.

This study set out to identify any barriers to acceptance that may arise from these factors, and others. To achieve this aim, we analysed semi-structured interviews from a sample (*N* = 30) of professionals and potential service users, using thematic analysis, in order to identify patterns in participants’ views and attitudes [55,56,57]. Several key areas relating to blockchain, in the context of healthcare, were covered, including:

- The nature and benefits of blockchain in healthcare;
- Internal and external attitudes towards the ideological and practical aims of delivering patient-centric data systems;
- Aspects of departmental/organisational culture that could hinder blockchain initiatives in the field of data ownership;
- Perceived barriers, either internal or external, to the development of blockchain applications;
- The implications of slow progress towards patient-centric data management systems.

The interviews were conducted in two phases:

- Structure and format: Prior to carrying out the interviews, primary open-ended questions were developed to provide the basis for follow-up. All questions were constructed in such a way as to avoid ‘leading’ the interviewee, which can result in bias.
- The interview processes: The time given to each interview was approximately the same (1 h). Consistency and validity were promoted through the use of field notes.

To help ensure that the proposed questions would deliver the depth and scope of the data required for the study [55,58,59,60], three pilot interviews were conducted before interviewing the full sample. The pilot process indicated that the relevant areas of investigation (described above) were adequately covered.

### 3.1. Sampling

This study employed a combination of purposeful and snowball [58,61,62] sampling techniques. Such a combination allowed the researchers to benefit from the advantages of both techniques [63,64,65]. While purposeful sampling was used to identify participants, who were highly informed on the topic in question, which helped to ensure that data was accurate and relevant, the snowball technique allowed the researchers to enlarge the sample to other, similarly expert, participants. This helped this study achieve richer and more comprehensive results.

The majority of the sample were professionals in the healthcare system with a clear perspective of the benefits and implications of introducing blockchain processes in delivering patient-centric systems, though it was also important to gather the views of other key stakeholders. Potential participants were as follows:

- Responsible for the development and/or management of administrative or clinical departments within national healthcare systems;
- Experienced in the design and implementation of blockchain projects;
- Other stakeholders in a patient-centric data management system (individuals not necessarily professionally involved with healthcare delivery).

The initial (purposeful) process of selecting participants was carried out by identifying appropriately qualified professionals on LinkedIn. After a comprehensive review of profiles, 70 invitations to participate were made, outlining the purpose of the study and emphasising its ethical construction. This resulted in 20 suitably qualified professionals volunteering to participate in the study. These professionals then recommended a further 10 potential participants. The final sample size was 30. All were based in GCC countries, and each country was represented by an (approximately) equal number of participants, in order to minimise the possibility of bias towards any country’s perspective (Table 1).

### 3.2. Data Collection

The 30 interviews which form the basis of this study were conducted over a two-month period. All original interviews were conducted in Arabic, and analysed in Arabic, with excerpts translated into English for the purposes of this report. Due to the diversity of geographic locations, interviews were carried out using online meeting platforms (Zoom, etc.) and were recorded using the platform’s tools.

All appropriate ethical guidelines were followed in conducting the research. Before each interview, the purpose and scope of the study were explained to the participant, and it was emphasised that there were no correct or incorrect answers to any question. Written assurances of full confidentiality were also provided, together with an explanation of how the data would be used. Each participant was informed that they could withdraw their participation at any time, and no part of any interview was recorded without full and explicit permission from the interviewee. All participation was voluntary, and no incentive, financial or otherwise, was offered to any participant.

Each interview contained 17 questions, and the process was meticulously designed to ensure that the questions were both rigorous and relevant to our study’s goals (all the questions are presented in Appendix A, together with a description of how the questions were designed). As the interviews were semi-structured, the questions were open-ended and conformed to a predetermined thematic framework [62]. This framework covered the broad areas described above. Example questions were the following:

- What benefits do you feel could be gained from deploying blockchain applications in healthcare?
- Do you feel that patient-centric data management is a desirable policy objective? Why?
- How realistic is any plan to implement patient-centric data infrastructures within your departmental framework?
- How do you think (health) service users will respond to patient-centric data management?
- Could failure to implement patient-centric data management have long-term consequences, either good or bad?

Following the interviews, each recording was matched against the field notes by two independent researchers. This helped to ensure the accuracy and consistency of data in preparation for the coding and analysis phase.

### 3.3. Data Analysis

As noted above, the study used thematic analysis [66] to identify themes in the interview data. The steps involved were as follows:

- Initial coding: Segment-by-segment coding to identify similarities in interview content.
- Focused coding: Codes thought to be of particular significance are grouped to form patterns.
- Theme search: Focused codes were recorded to facilitate the identification of groups that shared attributes of meaning. These sub-themes were then examined for higher-level commonalities to form main themes.
- Theme identification: This used (a) internal homogeneity (meaningful coherence within themes) and (b) external homogeneity (a clear and identifiable distinction between themes) to identify themes [58].

## 4. Results

As a result of the analysis, 4 main themes and 12 sub-themes connected to barriers to the adoption of blockchain (in healthcare) emerged (Table 2).

### 4.1. Administrative and Management

This theme contains four sub-themes. We will look at each in turn.

#### 4.1.1. Knowledge and Skills Recruitment

The participants expressed a wide variety of concerns about the specialist knowledge and skills required to successfully implement blockchain solutions. However, there was general agreement among all participants, including those not professionally involved in healthcare, that the advanced and immature nature of blockchain would represent a particular challenge in terms of recruitment. According to one participant, for example:

*Blockchain is still relatively new on the scene, and it’s a complex area. I realise that there are quite a few suitably qualified people around, but most of these have been swept up by private and high-paying enterprises. My guess would be that it would prove quite a challenge to find the number and type of staff required over the short or medium term.*

Another participant emphasised the difficulties of recruitment, saying:

*It will be tough enough to find the technical development staff needed, but there’s also the senior project management people to worry about. They need to be highly experienced in the strengths and limitations of blockchain technologies, and they’re few and far between at the moment.*

One participant, who is not a healthcare professional, commented:

*I’m not an expert in blockchain, but I know it’s what makes cryptocurrencies such as bitcoin work, so I would imagine there’s a high demand for blockchain specialists in the crypto sector. That might make it hard for the healthcare industry to find the right people.*

It is clear from these comments that the participants consider the practical challenge of system development to be a significant issue in itself, quite apart from any ideological or policy concerns.

#### 4.1.2. Funding and Financial Support

One sub-theme that was easily identified from the interview data was connected to cost. Several participants pointed out that blockchain projects are expensive to fund, and, as the technology is relatively new and untried in healthcare, projections of return on investment are not easy to make. Other interviewees mentioned that the nature of blockchain, which requires considerable computing power, could make systems expensive to run, so careful thought would have to be given to how they would be funded on an ongoing basis. A typical comment on this point was as follows:

*Using blockchain to build a patient-centric data management system sounds good idea on the face of it, but it could be surprisingly expensive to operate. A huge amount of information and a high blockchain transaction level is involved, which means it is likely to be very expensive to fund the consensus protocols and cryptographic needs of the underlying chain.*

Several participants emphasised the cost element of the recruitment difficulties discussed in the skills section above. For example:

*Specialist developers in DLTs [Distributed Ledger Technologies] such as blockchain are becoming more common, but they are still relatively rare animals. Most of them are either working in research, or are in relatively high paying posts with private companies. Health authorities will need to be prepared to pay high rates, if they want properly qualified staff.*

Other interviewees pointed out the implications of immature technologies such as blockchain. One said:

*You have to remember that, as blockchain is still relatively new and developing, the initial cost of the project is just the start. After that, there’s likely to be an ongoing requirement for infrastructure upgrades as the technology improves, as well as the addition of new features and functions to meet the needs of the healthcare system as societal requirements change.*

Overall, it seems that the nature of the technology, together with the relative scarcity of qualified project managers and developers, makes cost a significant issue.

#### 4.1.3. Management Commitment

Despite its many benefits, the nascent and unproven nature of blockchain technology is the source of significant levels of concern at the level of senior management and policy makers. But without a clear and positive commitment at these levels, to both the concept of patient-centric systems and the underlying blockchain technology, adoption of the technology is likely to be slow. As one interviewee put it:

*Projects such as that under discussion require buy-in across the board, and this simply won’t happen unless there is unequivocal backing from senior management. Personally, I don’t see that backing from my own employer, but my experience may not be typical.*

In fact, this view *was* quite typical, as several participants expressed a similar opinion, though some were more explicit about the causes of management concern. For example:

*There is certainly a lot of management hesitancy about moving forward with blockchain, and I think a lot of this is connected to personal risk. New technologies like this can easily fail due to unexpected problems, and the cost of project failure, however that’s defined, can be high at a political, institutional and personal level. Few senior managers are willing to take that risk.*

Other participants supported this view. One commented:

*I think that management, in general terms, is playing it safe at the moment. The stakes are high, and they’re waiting to see how other initiatives work out and whether they can learn from them.*

#### 4.1.4. Security

Although the consensus mechanisms and decentralised nature of blockchain mean that records are immutable and tamperproof, there remain questions over security, at both the institutional and individual levels, which can represent significant concerns to management and authorities. Nearly all of the participants felt that security, in one way or another, was a serious issue for the concept of patient-centric data management systems. According to one participant, for example:

*Despite blockchain’s famously high security levels, many people would be surprised at how vulnerable it can be. 51% attacks, for example, are well known in the Bitcoin arena and they can happen in any blockchain context. These give cybercriminals control over the system, which could be a disaster, particularly in a public service context.*

Another interviewee remarked:

*Because of a lack of standardisation, blockchain’s—and especially public chain systems—don’t necessarily meet all of the regulatory security and privacy requirements such as the GDPR and similar frameworks. I think this needs to be resolved before blockchain could safely be used in healthcare systems.*

While many participants commented on the institutional risks of blockchain, others pointed to risks at the personal stakeholder level. As one participant put it:

*Most members of the public will be unaware of the importance of protecting their private key. This means they could either mislay them or use weak keys—either way, it could lead to quite serious problems at a systemic and individual level.*

### 4.2. User Perspectives

This theme contains two sub-themes. We will look at each in turn.

#### 4.2.1. Ease of Use

While the majority of people today are comfortable with engaging with digital systems, there remains a very significant minority for whom digital technology is a challenge. Some do not engage at all. However, a healthcare system, by its nature, is fully inclusive. This means that, if the benefits of blockchain-based patient-centred systems are to be fully realised, they must be easy to engage with. This is not only true at the service user level, but at the clinical and administrative level also. However, many of the study’s participants expressed doubts as to whether the required levels of ease of use would be achieved. One participant said:

*In the end, a system like this is all about the patients. Unless they engage with it, it will fail. This means the interface must be ultra-easy to use, and access issues such as private keys must be simple to understand. My worry is, that a lot of people will find themselves unable or unwilling to use the system.*

Another interviewee made a similar point:

*Ease of use, in my mind, is critical. Unless the apps are simple and straightforward, engagement levels might be low. This would mean traditional record-keeping systems would have to be fully maintained, and that could negate, or significantly reduce, the benefits of a blockchain approach.*

While the importance of engagement of service users is critical, it is also essential that the system is accessible and easy for managers, administrators, and clinicians. The following remark by an interviewee was typical:

*It may be 2023, but a lot of professionals in healthcare are addicted to paper-based systems. The success of a patient-centric data management system will need something of a culture shift. For this to happen, the benefits will need to be clear, and the system extremely easy to use. I’m not convinced that either of those conditions would be true in the short term.*

#### 4.2.2. Privacy and Data Use

Administrative errors threaten the safety of thousands of patients worldwide every year. Although blockchain could realistically contribute to reducing these errors significantly, there remain a number of systemic security vulnerabilities. Some of these have been touched upon in the security subtheme (Section 4.1.1). However, there is a related, but separate, issue that was highlighted by many of the study’s participants. This issue is the problem of user perceptions of privacy and appropriate data use. As one participant commented:

*Although blockchain infrastructures offer high privacy levels, I would say that the large majority of the public don’t realise this. To them, an open system which gives them control over their own data could cause real concern. I think the authorities would have to run comprehensive educational campaigns to correct this impression, if people are to accept the system.*

The problem of inaccurate user perceptions, resulting from a lack of blockchain awareness extends to other areas related to privacy, such as data abuse. One participant, a professional outside of the healthcare field, commented:

*While, in reality, traditional record keeping systems can also be abused, there is a danger that the public would see a more apparently open system as presenting higher risk. This could present a problem in terms of adoption levels.*

This view was echoed by another participant, who commented:

*It’s ironic, really, that the privacy protections of blockchain, such as ZKPs [Zero-Knowledge Proofs], can deliver higher levels of privacy than anything we’ve known before, yet the average member of the public probably wouldn’t see it like that. I think there needs to be a broader understanding of how blockchain works before systems based on the technology will be accepted by the public.*

In summary, most participants felt that there was a wide gap between reality and public perception in terms of data privacy, and closing this gap represents a significant challenge.

### 4.3. Future Proofing

This theme contains four sub-themes. We will look at each in turn.

#### 4.3.1. Scalability

The scalability of blockchain networks is an important issue in many applications. This is particularly true with large public blockchains, which tend to have large amounts of data. This means there is a large number of transactions (changes to data). As each transaction must be validated through peer-to-peer verification, the network must have high scalability (the ability to support an increasing transaction load, as well as more network nodes). This represents a key challenge for the healthcare sector. One participant, for example, pointed out that:

*One problem is that the system under discussion is fundamentally public facing, which means it not only must be easy to use, but it has to be real-time. Unless users see changes pretty much instantly, they are unlikely to engage with the system. However, not all blockchains can deliver the required scalability.*

Another participant made a similar point:

*In theory, the system proposed could consist of small private blockchains, which would help to maintain scalability and speed. But this would be expensive. In practise, the chances are that scalability, or the lack of it, would present a major challenge to healthcare providers.*

Scalability could also present issues where payments are concerned, according to several participants. For example:

*In itself, transaction speed might not be a major problem in many contexts, such as data management. But the system will also need to allow for payment processes in some situations, such as private consultancies, and this could be a much more serious challenge. Scalability of the system will be essential.*

This sub-theme of the results highlights that, even though blockchain has been used in real-world applications for over a decade, scalability issues may still hinder growth and innovation.

#### 4.3.2. Interoperability

Historically, blockchain technology has evolved in a siloed environment, which has led to a number of issues that have hindered the adoption of blockchain. One of these issues is a lack of interoperability—different blockchains do not easily ‘talk’ to other systems. Several participants identified this as a potential problem. As one interviewee put it:

*It’s crucial that blockchains used in a healthcare system can interoperate, and communicate with legacy systems. As things stand, this ability is, though improving, still limited, and could present real challenges, in terms of enabling healthcare systems based on blockchain to adapt to future needs easily and cost effectively.*

Another participant emphasised the same point, but was a little more positive about emerging solutions:

*There’s no doubt that interoperability has been major factor in Decisions whether or not to implement solutions based on blockchain. It’s still an important factor, but at least realistic and viable solutions are now beginning to emerge, such as Over ledger from Quant [Network], and Ark.*

It is important to note that interoperability is not only about blockchain-to-blockchain communications. To be effective, blockchains must also communicate with other IT systems and applications within the healthcare infrastructure. This point was made by a number of participants. For example:

*If a patient-centric system is going to deliver on its potential, it needs to be able to exchange data with a wide range of other systems, internal and external. I’m not convinced that this ability exists right now, without compromising core attributes such as security and privacy.*

In short, while interoperability is improving rapidly, it is still a significant concern and may limit options in the future.

#### 4.3.3. Standardisation

Another significant issue is the lack of standards—each different blockchain has been developed with its own set of protocols. This has played a large part in inhibiting mass adoption. One participant remarked:

*Standards are necessary for any technology to succeed at a global, or even national, level. This is no different for blockchain. It’s true that the landscape is beginning to change, But standards take time to develop and we’re not there yet. This means that there are real risks involved with Investing huge amounts in blockchain-based healthcare systems.*

The observation that blockchain standards are beginning to emerge was made by several participants. However, most still had caveats. According to one:

*It’s good to see that several industry alliances and other bodies are now collaborating to create global blockchain standards, but we’re still some way away from a framework that will trigger mass adoption of the technology.*

The issues of standardisation and interoperability, though separate, are also closely connected. As one participant said:

*It’s true that blockchain technology offers a range of major benefits over other data exchange solutions, but the lack of industry standards could cause serious interoperability issues.*

#### 4.3.4. Sustainability

Most blockchains are energy intensive to run. The implementation of systems based on the technology may therefore not be consistent with the commitment of most countries to become environmentally sustainable and energy efficient. For example, all 193 members of the UN’s Sustainable Development Goals (SDGs), which include climate and clean energy targets for 2030. This issue was a clear concern among the study’s participants. One interviewee, for example, commented:

*The energy consumption issue is a real dilemma. Most blockchains operate on a proof-of-work basis which needs a lot of energy, which can be hard to justify, given current concerns over climate change and so on. The alternative is proof-of-stake models, but although they’re less energy-intensive, they’re much more complex and can lead to system vulnerabilities.*

The above comment was repeated in various forms. For example, another participant said:

*Implementing blockchain could easily end up being a decision that the authorities might regret, due to the energy demands of the system. I seem to remember that it was the power requirements of blockchains that led to Tesla reversing their decision to allow payment with Bitcoin in 2021. That surely tells us something about the difficulties involved.*

Another participant recognised the sustainability issue, but was more positive:

*Some experts think that the types of blockchain suited to use in healthcare have far lower energy requirements than those of Bitcoin. But, even if this is the case, combatting the negative perceptions of blockchain energy consumption won’t be easy.*

The technology is evolving rapidly, and it remains to be seen whether healthcare blockchains will encounter energy usage issues. If suitable solutions are identified, it will be imperative to combat the negative perceptions of current blockchain energy consumption.

### 4.4. Regulatory Issues

This theme contains two sub-themes. We will look at each in turn.

#### 4.4.1. Compliance

The implementation of blockchain in healthcare must comply with relevant national, and in some cases, regional (e.g., GCC, EU, US) regulatory frameworks. These frameworks vary according to country/region and therefore make different demands of the system. Due to blockchain’s relatively immature technical nature, compliance is not always easy to ensure. This was highlighted by several participants. For example:

*I believe most GCC countries now have an equivalent to the EU’s* [European Union] *GDPR* [General Data Protection Regulation], *which contains a right to be forgotten. However, this is incompatible with blockchain’s immutability. I’m not sure how this will be resolved.*

Another participant said:

*While most regulatory frameworks don’t yet have a standard for decentralised identity, this is something that will almost certainly emerge soon. But until we know its precise form, it will be difficult to know how easy compliance will be. It might require processes within the blockchain system to be reengineered.*

The problem of complying with the requirements of most regulatory frameworks to anonymise personal (sensitive) information was raised by several interviewees. For example:

*The main difficulty is establishing whether the information held on the [blockchain] system is considered sensitive. And even when this is known, there are question-marks over whether the information can be anonymised in a way that’s compliant with regulations such as GDPR.*

#### 4.4.2. Governance and Liability

It is important to establish a clear and robust governance model concerning interactions between parties involved with the blockchain network. In a healthcare system, these parties would include the network operator and its participants. While this is not, in general terms, especially difficult in a technical or legal sense, the relatively novel nature of blockchain could present a challenge. As one participant phrased it:

*Blockchain poses a range of different risks connected to issues such as security, confidentiality and data protection. However, these risks aren’t, as yet, fully understood in practical context, so the attribution of liability needs to be carefully analysed.*

Another participant made a similar point:

*It is important to establish the legal structure, liability and governance model of the blockchain systems, so that all rules, rights and obligations are legally clear. This is critical to ensuring everyone involved, from service users to administrators can use the system without concern.*

While most blockchain healthcare systems will fulfil internal national needs, there are situations where they may be required to function across borders. This could pose a number of complex jurisdictional issues which require careful consideration. One participant said:

*If blockchain nodes are located in different countries or regions, it’s not clear which legal framework would be relevant. Local laws may apply, but there is some confusion over exactly what laws would be enforceable and how they would be enforced.*

### 4.5. Comparison of IT and Clinical Perspectives

A further analysis of the data allows for a comparison between the perspectives of the IT specialists whose focus is on the management and delivery of IT systems and processes and the clinical practice managers whose focus is on clinical provision. This is based on the themes and sub-themes identified through the analysis, which are then traced through the data. This enables us to list concerns and prospects recognised by these two groups, which are related yet importantly different in certain respects, as follows.

#### 4.5.1. IT Specialist Perspective

- Scalability: IT specialists must consider how well a blockchain solution can scale to support a large number of transactions. For example, public blockchains like Bitcoin and Ethereum can process a limited number of transactions per second, which may not be suitable for a large healthcare organisation. Private or consortium blockchains, on the other hand, might provide better scalability.
- Integration with Existing Systems: IT specialists have to ensure that the blockchain solution can integrate seamlessly with existing health IT systems. This involves considering issues like data migration, user training, and system maintenance.
- Regulatory Compliance: IT specialists must ensure that the blockchain solution complies with regulations related to health data, such as the Health Insurance Portability and Accountability Act.
- Blockchain Type: IT specialists need to decide between public, private, or consortium blockchains. Public blockchains are open to anyone and are secured by decentralization, but they might not be suitable for sensitive health data due to privacy concerns. Private and consortium blockchains, which are only accessible to invited participants, might be a better choice for healthcare applications.
- Smart Contracts: IT specialists are likely to be interested in the potential of smart contracts, which are self-executing contracts with the terms of the agreement directly written into lines of code. In healthcare, smart contracts could automate many processes, such as claims adjudication in health insurance.
- Data Standardisation: To ensure interoperability, IT specialists need to consider standardising the data stored in the blockchain.

#### 4.5.2. Clinical Practice Manager Perspective

- Patient Engagement: Clinical Practice Managers are likely to view blockchain as a tool for enhancing patient engagement. For example, blockchain could enable patients to control who has access to their health data, thereby promoting a more patient-centric approach to healthcare.
- Collaboration with other Healthcare Providers: Blockchain could facilitate collaboration by creating a shared, immutable record of patient data that can be accessed by different healthcare providers. This could lead to more coordinated and efficient patient care.
- Cost Implications: Clinical Practice Managers must consider the cost implications of implementing blockchain technology. This includes not only the costs involved in the technology itself, but also training costs, maintenance costs, and potential cost savings from improved efficiency.
- Staff Training and Adaptation: Clinical Practice Managers would need to plan for staff training to ensure that all staff members understand how to use the new system.
- Patient Empowerment: Clinical Practice Managers may see blockchain as a way to empower patients. With blockchain, patients could have more control over their health data, deciding who can access it and what they can do with it.
- Improved Care Coordination: Blockchain could improve care coordination by providing a single, up-to-date, and immutable record of a patient’s health history. This could help all providers involved in a patient’s care stay on the same page.
- Operational Efficiency: Clinical Practice Managers may be interested in how blockchain could improve operational efficiency. For example, blockchain could speed up the claims process in health insurance by reducing the need for intermediaries.
- Change Management: Implementing a new technology like blockchain would involve significant change. Clinical Practice Managers would need to manage this change carefully, ensuring that staff are trained, also that relationships with external agencies and providers are developed and maintained, that patients are in certain respects trained, informed, and appropriately advised, and that workflows are updated as necessary.

#### 4.5.3. Reflection

To aid the process of consolidating these outcomes, a further exercise was conducted in which an IT specialist and a practice manager were interviewed and encouraged to reflect further on the issues, on the nature and sources of their expectations surrounding blockchain technologies, and on underlying factors critical to success.

They emphasised that the majority of clinical practice managers typically undergo a profound process of culture change management. This process, which often occurs before, during, and after the implementation of new systems or practices, significantly shapes their comprehension of and approach to implementing novel technologies or changes. Their perspectives are formulated in the context of a variety of factors, including their hands-on professional experiences, continuous training and personal development, interactions with colleagues and industry experts, and adherence to regulatory standards and guidelines.

In the context of healthcare settings, IT professionals serve as a pivotal force in evaluating and deploying new technologies such as blockchain. These professionals are equipped with the technical expertise to decipher the complexities of blockchain technology, its potential applications, and the prerequisites for its successful implementation.

The successful integration of blockchain technology within healthcare systems is not a task that rests solely on the shoulders of IT professionals. It demands a concerted and cooperative effort from clinical practice managers, IT professionals, and other significant stakeholders. Clinical practice managers bring to the table indispensable insights into the functional needs of the clinic. Their understanding of day-to-day operations, patient interactions, and workflow dynamics is critical to shaping the implementation strategy.

On the other hand, IT professionals employ their specialist knowledge to architect the most effective technical solutions that meet these needs. Their expertise ensures that the blockchain technology is tailored to fit seamlessly within the existing system, enhancing efficiency without disrupting established workflows.

The professionals see this harmonious collaboration as the backbone of a successful implementation strategy. It ensures that blockchain technology is not just technically integrated, but also woven into the fabric of the clinic’s operations in a manner that maximizes benefits, to serve the clinic and its patients optimally.

Overall, we seem to observe here that the complementary concerns of these professional groups emphasise the need, above all, for clear articulation and excellent communication between them. This will be especially critical as the implications of introducing a new technology become clearer. For example, it was remarked that the technology should enhance efficiency “without disrupting established workflows”; but, in general, it cannot be expected that established workflows will, or always should, remain unaffected. This may well at times be inconsistent with the goal of enhancing efficiency. Working out what kinds of changes will be inevitable, which should be embraced and which should be resisted, is a process that strongly depends on the collaboration, but may test the harmonious relationship quite severely.

## 5. Discussion

This study has explored the barriers to the use of blockchain-based infrastructures in healthcare, with particular emphasis on the delivery of patient-centric data management systems, which leads to a focus on issues such as data ownership and privacy. A sample of 30 professionals (healthcare and non-healthcare) were interviewed, and the resulting data was subjected to thematic analysis. In total, 12 specific themes relating to barriers were identified, categorised under four main themes: Administrative and Management, User Perspectives, Future Proofing, and Regulatory Issues. In light of these themes, a comparison was carried out between the perspectives of professionals from the IT domain, on the one hand, and clinical practice management on the other.

The results of this study identified two main themes which were of equal concern to the participants: administrative and management issues, and future-proofing issues (Table 2). All of the sub-themes that were identified within these main themes are common to any kind of information technology that might be used in this area, but assume particular forms and significance in relation to the (relatively) new and unproven nature of blockchain technology. This is a result of the fact that, while blockchain is widely recognised as having the vast potential to drive social and economic development, by revolutionising many business and public sector processes, it remains a developing technology and raises questions about many of these key issues, and in particular security risk, cost, scalability, stability and flexibility [43,67]. This study also found that issues connected to end-user perceptions and regulation were also a concern. For example, most participants felt that the public perception of data privacy was significantly different from the reality, and this gap would not be easy to eliminate. This finding reflects those of a number of other studies, such as in [68,69].

On some barriers, such as Knowledge and Skills Recruitment, there was particularly strong agreement. Such a consensus suggests that the practical issue of system development will be a significant challenge, separate from any political or doctrinal concerns. This is consistent with the findings of other studies and relevant skills and recruitment sources. Although a similar issue could arise in relation to other technologies, it is apparently perceived as especially serious for the case of blockchain technologies, due to the expected competition for skills from areas such as cryptocurrencies.

Another area in which the participants were in strong agreement was management commitment. A lack of commitment at senior levels will inevitably have a negative impact on the adoption levels of any technology [70,71], and this lack of support for blockchain technologies was highlighted by the majority of participants. This view is supported by some other sources [72], who argue that there are often powerful vested interests that oppose the wider developmental impact of blockchain technology and may do everything in their power to hinder its implementation.

However, the general evidence from the wider field of literature is not in total agreement with these observations. While there is some evidence that senior clinicians and other medical personnel are sceptical of the technology [73,74], there is a considerable amount of de facto evidence that senior tiers of management and policymakers are strongly in favour of adoption—this evidence lies in the fact that blockchain is being increasingly deployed in the healthcare sector [75,76]. However, this evidence from the literature applies only to the general field of healthcare, not to the specific use case of patient-centric data management systems. Further, all participants in the current study were from GCC countries, and cultural and political factors may have played a role in biasing the results. Further research is needed here.

Some sub-themes in the study showed a divergence of views. The barrier of interoperability, for example, was seen by all participants to be a major ICT development issue, but some participants saw it as insurmountable over the short and medium term, while others saw it as less critical, as there is evidence of viable solutions already emerging. The literature is inconclusive on this point. Interoperability is often difficult even in cases where there are mature standards, due to differences in implementation and the problems of updating as technologies and standards evolve; the present state of blockchain technology does not make this any easier. However, while there is a broad consensus that blockchain interoperability remains a key issue [77], there are mixed views on the degree to which it is a problem. There are now several examples of large-scale, real-world blockchain projects that depend critically on interoperability, such as LACChain, which is a pan-regional blockchain programme across 12 countries in Latin America and the Caribbean, which are designed to drive social and economic development, promote financial inclusion and sustainability, and create new efficiencies through digitisation [78]. In this case, interoperability of multiple blockchains has been provided through a solution from Quant Network—a solution that is ‘blockchain agnostic’ and can be applied to a wide range of industries, including healthcare. However, there is also an argument that true interoperability between blockchains is impossible [79] and that the solutions mentioned must therefore relax the formal definition of a blockchain in some way. It remains to be seen whether such a relaxation may be shown to be of particular concern in a healthcare data context.

Standardisation, which is a related but separate issue to interoperability, was also identified as a barrier by the participants, though several observed that various standards bodies are beginning to develop international frameworks. Nonetheless, the lack of standardisation remains an issue that inhibits the development of blockchain-based systems in healthcare, as well as other sectors. This is because effective healthcare, and particularly patient-centric data management, can only be delivered through the integration and data sharing of multiple different ecosystems and legacy systems, and this requires the development and implementation of middleware and interfaces [45,80,81]. This development process can only be effective if the blockchain platforms are developed to a common set of standards [82]. One can observe that organisations such as the Institute of Electrical and Electronics Engineers (IEEE) [83] and the European Commission [84] are working to develop and promote standards as quickly as possible, but their uptake and overall effectiveness are not yet clear.

Another issue that was identified as a barrier, but with different degrees of resolvability by the participants, was scalability. Although many participants saw scalability as a barrier to be addressed if blockchain is to fulfil its developmental potential, some pointed out that if a system is built around private blockchains, then it could be less of an issue [85]. Other interviewees, however, remarked that the use of private blockchains is more complex and expensive, and so may prove impractical in many contexts. Overall, the study confirmed the findings of other research that has looked at the issue of scalability in more general health (and other) contexts [86,87].

Because of the nature of the kind of development under discussion in this study (patient-centric data management), security is a particular concern. Nearly all of the participants felt that, despite the inherent security strengths of blockchain, this remained a serious issue. This reflects the findings of other studies, which have highlighted that blockchain infrastructures are vulnerable [88,89]. One participant mentioned the possibility of 51% of attacks, which are recognised as a significant risk [90]. However, we note that the nature of the risk is not necessarily clear. In practice, these attacks, along with Sybil attacks, seem to be a risk to the integrity of the organisation of the blockchain; they might allow arbitrary data to be added or removed, and thus completely disrupt the operation of systems. This may not, e.g., allow the attacker to read any personal patient-related information directly (since such information is usually stored off the blockchain), but may allow it to be accessed indirectly if the blockchain controls access to the patient data. We conclude that security, and in particular the perception of security and risk, is among the main concerns/barriers that need to be addressed.

Today, the issue of environmental sustainability forms a key part of the CSR (Corporate Social Responsibility) policies of nearly all organisations. It is therefore a critical consideration for healthcare authorities. However, most blockchains are energy intensive to run, which has implications for sustainability. In fact, it has been reported that in some sectors, such as education, lack of sustainability is the prime reason for low adoption rates of blockchain [42]. The results of this study indicate that it remains a key development issue in healthcare, although some participants were of the view that solutions were emerging. It should be noted that this study was confined to the implications of blockchain for environmental (as opposed to social and economic) sustainability—future studies on its implications for social and economic sustainability development would be useful.

In any context, the issues of regulatory compliance and governance are important. But they are particularly important in public service contexts such as healthcare. In general terms, this may be considered a complex, but uncontentious, issue. However, the relatively novel setting of blockchain applications presents significant challenges, as there is currently no ‘settled law’ of blockchain [12]. In other words, regulatory frameworks and national/international governance models are yet to be fully defined. This issue was stressed by several participants in the current study.

Overall, the result of this study suggests that the barriers to implementing a blockchain-based, patient-centric data management system fall into two categories: those that were supported by all participants as being a major challenge to the adoption of the technology, and those which, while still a significant challenge, are seen as possibly having viable, short-term solutions. For example, issues such as skills recruitment and management commitment were shown to be ongoing problems without an obvious or immediate resolution, while it is believed that interoperability and scalability can currently be addressed, at least to some extent. This has been demonstrated by use cases in a number of sectors, including healthcare [86,87].

Our comparison of perceptions among different groups suggests that their concerns tend to be complementary: one way of looking at this is that they worry about the things that fall into their own area of responsibility and can do something about them, while they leave other concerns to others. This is a rational approach, but without excellent lines of communication may carry the risk that issues are addressed without full consultation, leading to conflict, dissatisfaction, loss of trust, and potentially a fall in the level of acceptance of the whole technology strategy.

## 6. Conclusions

Today, improving healthcare for citizens is a key development policy objective of countries around the world. One route to achieving this, which is attracting attention from an increasing number of governments, is to change the data ownership model by transitioning to a patient-centric system. Such an approach can not only improve patient outcomes and satisfaction, but increase the efficiency of healthcare resources. However, the design and delivery of such systems is a complex issue. While a possible solution is to deploy blockchain technology, this is still in its relatively early stages of development. Its application can present significant challenges to ICT development and introduction—challenges that differ from use case to use case.

This study seeks to identify the key barriers to the acceptance of blockchain-based, patient-centric data management systems, as an important first step in achieving widespread adoption. Unlike the few existing studies on the topic, this research examines the perspectives of professionals (healthcare and non-healthcare) in order to identify and prioritise relevant challenges. Our approach addresses a research gap identified by existing reviews [21]. The results are expected to be of interest to healthcare bodies looking to improve the effectiveness and efficiency of their service delivery infrastructures.

This study identified 12 barriers, which were classified into 4 main themes (Administrative and Management, User Perspectives, Future Proofing, and Regulatory Issues). Of these main themes, two (Administrative and Management and Future Proofing) were of most concern to participants, though the sub-themes (barriers) within these main themes varied in their significance and potential impact on blockchain adoption. Some of the barriers were perceived by participants to have no short-term solution, while others, though still significant, had short-term workarounds or resolutions. Overall, the two most challenging barriers for the immediate term were found to be (a) skills recruitment and (b) management commitment. Any of these barriers will need to be addressed in an environment of full and open consultation across different stakeholder groups, to promote and enhance a “harmonious collaboration” that will be crucial to maintaining acceptance across the organisation.

These findings have some important theoretical and practical implications for GCC healthcare systems. GCC countries are, for example, actively working to improve their healthcare infrastructures, and the deployment of distributed ledger (blockchain) technology could play a key role in achieving objectives such as improved outcomes and system efficiency. However, the adoption of such technology by users is critical to success, so understanding barriers to acceptance is key. The identification of these barriers will not only help policymakers and healthcare providers transition to blockchain-based systems effectively, but will also help to ensure that new systems and processes are fully aligned with the needs and expectations of GCC populations.

We believe that our study will contribute significantly to the understanding of the issue of acceptance barriers and help pave the way for more effective implementation strategies for blockchain technology in the region’s healthcare sector.

It is important to bear in mind that the study reported here was carried out in the GCC countries. These all have advanced healthcare systems, comparable with those found in other developed countries, but the cultural context varies in some respects, and this could affect the perceptions of participants. Other research has noted cultural differences in this region in, for example, attitudes to information privacy, data, and security, and in approaches to these issues among management and policy makers [91,92,93]. We believe that many of the findings we report would be similar in other advanced healthcare contexts. A fuller comparative study would be extremely valuable but, as yet, the literature does not offer comparable findings for other contexts. Our study is necessarily limited, but we hope to inspire and inform further research that will allow investigation of potential regional or cultural specificities in more detail.

Although the study sought to obtain generalisable conclusions through its methodology and sampling, it should be noted that the sample (interviewees) consisted of professionals from GCC countries, and therefore may have been affected by cultural issues. Further research with a broader sample would provide further insights. Our current study focused on qualitative analysis to explore the barriers to acceptance of blockchain-based patient-centric data management systems in healthcare, and we believe this is an essential precursor to potential further study using a more quantitative methodology. Statistical data needs to be acquired and interpreted in a context where, for example, questionnaires can be designed with a clear view of how participants will understand them. Well-informed quantitative research can then provide insights with potential value to explore comparative analysis across different societies and cultures.

## Figures and Tables

**Table 1:** Summary of details of interviewees.

Participant No.	Job Title	Years of Experience	Country
P1	Professor of Cryptology	16	UAE
P2, P3	Academic Advisor in Blockchain	6	Bahrain
P4	Post-doctoral Researcher in Blockchain Systems	4	Bahrain
P5	Clinical Practice Manager	13	Oman
P5, P6	Lecturer in Information Technology	11	Saudi
P7	Professor in Computer Engineering	7	Saudi
P8	Multidisciplinary Team Leader in Healthcare	6	Oman
P9	Clinical Practice Manager	8	UAE
P10	Hospital CEO	10	Bahrain
P10, P11	Pharmaceutical Product Manager	7	Kuwait
P12	University IT Developer	5	UAE
P13	Healthcare Manager	3	Qatar
P14	Clinical Operations Manager	3	Kuwait
P15	Project Manager, IT	4	Saudi
P16	Professor in Informatics	14	Oman
P17	Associate Professor in Software/IT	7	Oman
P18	Ph.D. Student in Distributed Ledger Technology	4	Qatar
P19	Clinical Operations Manager	11	UAE
P20, P21	Senior Healthcare Scientist	12	Saudi
P22	Professor in Computer Engineering	7	Kuwait
P23	Professor of Blockchain Technology	5	Qatar
P24	Professor in Informatics	14	Saudi
P25, P26	Director of Healthcare Services	12	Bahrain
P27	University IT Manager	4	Kuwait
P28	Director of Healthcare Services	10	Saudi
P29, P30	Hospital CEO	17	Qatar

**Table 2:** Main themes and sub-themes.

Main (Barrier) Themes	Sub-Themes
Administrative and Management	Knowledge and Skills Recruitment
	Funding and Financial Support
	Management Commitment
	Security
User Perspectives	Ease of Use
	Privacy and Data Use
Future Proofing	Scalability
	Interoperability
	Standardisation
	Sustainability
Regulatory Issues	Compliance
	Governance and Liability

## Data Availability

Data are available on request due to restrictions of privacy.

## Appendix A

Interview Questions: As is conventional for semi-structured interviews, this study’s interviews were designed with open questions to provide the researcher with the opportunity to explore particular themes or responses further. Much consideration was given to the question design, to ensure that they provided information that was relevant to the study’s goals. The key guiding principles of interview design were as follows:

Consistency with Research Objectives: Every question was designed to help identify and understand the barriers to the acceptance of blockchain-based patient-centric data management systems.

Theoretical Relevance: All questions were designed to leverage the existing knowledge base (from the current literature) to ensure they were consistent with a solid theoretical framework built around general factors such as technology adoption and resistance to change, as well as specific factors such as data management using blockchain systems.

Expert Assessment: Before being used in interviews, all prototype questions were assessed by several experts with experience in blockchain technology, healthcare systems, and the cultural/regulatory landscape of the GCC. This was important in ensuring that all questions were clear, relevant, and likely to provide the richness of response required by the study’s analysis.

Pilot Test: Before carrying out actual interviews for the study, several pilot interviews were conducted to test clarity and quality of response. Following these pilot interviews, a number of refinements were made, in order to remove potential ambiguity.

The researchers believe that the rigorous process described above resulted in a set of questions that yielded valuable contributions to answering the study’s RQ.

The questions used in the interviews were as follows:

1. What are the key benefits of blockchain technology for healthcare?
2. How can blockchain be used to improve the security of patient data?
3. How can blockchain be used to improve the interoperability of patient data?
4. How can blockchain be used to improve the efficiency of patient care?
5. How can blockchain be used to improve the transparency of patient care?
6. How can blockchain be used to improve the accountability of healthcare providers?
7. What are some specific examples of how blockchain is being used in healthcare today?
8. What are the challenges of deploying blockchain applications in healthcare?
9. How do you think blockchain will impact the future of healthcare?
10. What is patient-centric data management?
11. What are the benefits of patient-centric data management?
12. How can policy be used to promote patient-centric data management?
13. What are some specific examples of how policy has been used to promote patient-centric data management?
14. Do you think patient-centric data management is a desirable policy objective? Please give reasons for your answer.
15. What are the challenges of implementing patient-centric data infrastructures within a healthcare department?
16. What are the benefits of implementing patient-centric data infrastructures within a healthcare department?
17. How would you go about implementing patient-centric data infrastructures within a healthcare department?
